# Topological Analysis of Small Leucine-Rich Repeat Proteoglycan Nyctalopin

**DOI:** 10.1371/journal.pone.0033137

**Published:** 2012-04-02

**Authors:** Pasano Bojang, Ronald G. Gregg

**Affiliations:** 1 Department of Biochemistry and Molecular Biology, University of Louisville, Louisville, Kentucky, United States of America; 2 Ophthalmology and Visual Sciences, University of Louisville, Louisville, Kentucky, United States of America; University of Cambridge, United Kingdom

## Abstract

Nyctalopin is a small leucine rich repeat proteoglycan (SLRP) whose function is critical for normal vision. The absence of nyctalopin results in the complete form of congenital stationary night blindness. Normally, glutamate released by photoreceptors binds to the metabotropic glutamate receptor type 6 (GRM6), which through a G-protein cascade closes the non-specific cation channel, TRPM1, on the dendritic tips of depolarizing bipolar cells (DBCs) in the retina. Nyctalopin has been shown to interact with TRPM1 and expression of TRPM1 on the dendritic tips of the DBCs is dependent on nyctalopin expression. In the current study, we used yeast two hybrid and biochemical approaches to investigate whether murine nyctalopin was membrane bound, and if so by what mechanism, and also whether the functional form was as a homodimer. Our results show that murine nyctalopin is anchored to the plasma membrane by a single transmembrane domain, such that the LRR domain is located in the extracellular space.

## Introduction

Nyctalopin is a small leucine rich repeat containing protein that is required for normal vision [1], [2] and is localized to the dendritic tips of depolarizing bipolar cells (DBCs) [3]. It is predicted to be a member of the small leucine rich proteoglycan (SLRP) family (for review see [4]). The core of nyctalopin consists of eleven leucine rich repeats (LRRs) that are capped at the N-terminus and the C-terminus by cysteine rich motifs. The consensus LRR is 24 amino acids with the sequence, x-x-I/V/L-x-x-x-x-F/P/L-x-x-L/P-x-x-L-x-x-L/I-x-L-x-x-N-x-I/L, where x is any amino acid, and was initially identified in the human alpha 2-glycoprotein [5]. The N- and the C-terminal caps have a consensus arrangement of Cx_3_Cx_3_CxCx_6_Cx_3_C and CCxCx_19_Cx_23_C, respectively. Each tandem LRR domain is folded into β-sheets and α-helices joined by loops. This arrangement of β-sheets and α-helices gives the tandem LRR domain a horseshoe shape with parallel β-sheets lining the concave side and α-helices lining the convex side (Figure S1). At the N-terminus of nyctalopin there is a predicted signal sequence. At the C-terminus of human nyctalopin there is a consensus sequence for addition of a glycosylphosphatidylinositol (GPI) anchor [1], [2]. However, in mouse this site appears to be absent, rather there may be one or more transmembrane domains [6].

When expressed in heterogeneous expression systems, both human and murine nyctalopin were determined to be anchored to the cell surface [7], [8]. Phosphatidylinositol-phosphalipase D (PI-PLD), which specifically cleaves GPI anchors, was able to release human nyctalopin from the cell surface, but not mouse nyctalopin [7]. In addition, hydrazine, which is an inhibitor of GPI cleavage and forms complexes with GPI anchored proteins, does not complex with murine nyctalopin. These data suggest that murine nyctalopin is anchored to the cell surface by a mechanism other than a GPI anchor, possibly via transmembrane domains.

The predicted signal sequence in nyctalopin indicates it is likely processed by a co-translational mechanism. Co-translational targeting is mediated by the ribonucleoprotein complex (RNC), the signal recognition particle (SRP) and its cognate membrane-associated receptor (SR) located on the ER (reviewed in [9], [10]). Membrane proteins are inserted into the ER membrane either as type I or type II membrane proteins. Type I and II membrane proteins have their N-terminus located in the ER lumen or the cytoplasm, respectively. The orientation in the membrane of the first transmembrane domain is determined by three factors. First, proteins with stable N-terminal tertiary structures tend to stay in the lumen of the ER because they are too large to traverse the translocon [11]. Second, the charge distribution either before or between the transmembrane domains are important (reviewed in [12]). If the region is positively charged then the intermembrane region tends to remain in the cytosol. Third, longer hydrophobic regions favor localizing the N-terminus in the lumen of the ER [13], [14].

Once translation and membrane insertion is complete in the ER, the proteins are sorted and transported to the appropriate sub-cellular compartment using a complex series of events that occur in the Golgi network. Trafficking of the proteins from the ER to the Golgi relies on the coatomer protein complex II (COPII) and the adaptor protein (AP) – clathrin complexes (AP-clathrin complexes) (reviewed in [15], [16]. Transport of proteins from the Golgi to the plasma membrane or the endosomes is done by vesicle budding of the Golgi and fusing to the plasma membrane (reviewed in [17]).

SLRP family members have diverse membrane orientation and sub-cellular localization, which reflects their functional diversity. Some members such as the TrK family of nuclear receptors are integral membrane proteins [18]. Others, like *Drosophila* connectin are GPI anchored [19] and the ribonuclease inhibitors are localized to the cytoplasm [20]. In addition, solution X-ray scattering experiments show that both decorin and biglycan are dimers in solution and crystal structures predict that they form dimers via interaction through their concave faces [21], [22], [23]. Gel filtration chromatography, light scattering and sedimentation equilibrium experiments indicate opticin also forms dimers [24]. These data suggest that the biologically active form of decorin, opticin and biglycan may be a dimer.

In this report, we used a combination of yeast two-hybrid and *in-vitro* translation approaches to investigate whether murine nyctalopin is oriented with the N-terminus in the extracellular space and if it is anchored to the membrane by a single transmembrane domain. We also examined whether nyctalopin could form homo-dimers in yeast.

**Table 1:** Transmembrane (TM) domain predictions for murine nyctalopin.

				Location of TM		
Program	#TM(s)	C-terminus	N-terminus	TM3	TM2	TM1
HMMTOP2.0	3	Out	In	260–278	309–328	452–473
TMPred	2	In/Out	In/Out	None	309–329	453–473
TopPred2	2	In/Out	In/Out	None	309–328	455–471
SOSUI-Mp1	1	In	Out	None	None	452–473
MemPype	1	In	Out	None	None	452–472
TMHMM2.0	2	Out	Out	None	309–329(0.6)	455–473(0.9)
DAS	0	None	Out	None	None	None

**Figure 1:**
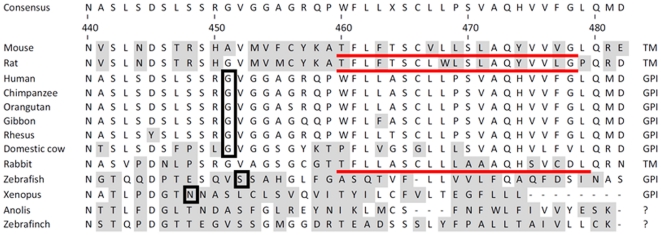
Sequence alignment of the carboxy termini of nyctalopin from several species. Predictions are based on data from the MemPype prediction site (http://mu2py.biocomp.unibo.it/mempype). Putative transmembrane domains are underlined and the amino acids to which the GPI is predicted to be attached are boxed. Shaded amino acids are those that differ from the consensus sequence. The most likely mode of membrane anchoring is indicated on the right side of the figure. The alignment was created using ClustalW implemented in the Lasergene software package (DNAStar Inc, Madison, WI). Accession numbers of sequences used for the alignment were : Mouse, AAM47034; rat, NP_001094437 ;human, AAG42685; Chimpanzee, XP_001138632 ; Orangutan, XP_002831599.1; Gibbon, XP_003271059; Rhesus monkey, XP_001087613; Domestic cow, XP_002700268; rabbit, XP_002719884; Zebrafish, ABB03696; Xenopus, NP_001087744; Anolis, XP_003219032; and zebrafinch, XP_002190541.

## Results

### Topology of Murine Nyctalopin

Nyctalopin was predicted to be bound to the membrane by a GPI anchor in human [1], [2] and have two transmembrane domains in rodents [6]. Expression in cultured cells provided some experimental support for these predictions, although the mechanism and orientation of murine nyctalopin was less certain [7], [8].

**Figure 2:**
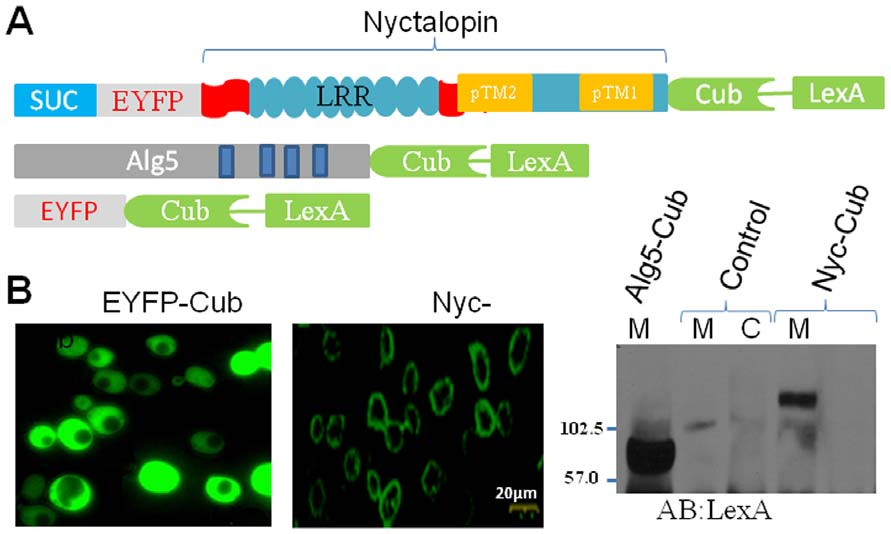
Nyctalopin is membrane bound in yeast. A. Schematic diagram of constructs used to determine the localization of nyctalopin in yeast. Nyc-Cub (109.4 kDa) is a fusion protein with the yeast invertase signal sequence (SUC) (20aa), EYFP (217aa), nyctalopin (453aa) and the Cub-LexAVP16 artificial transcription factor (350aa). Alg5-Cub (77.5 kDa) is a yeast ER membrane bound protein fused to Cub-LexAVP16 and is used as a positive control. EYFP-Cub has EYFP fused to Cub-LexAVP16. **B.** (Left) Confocal images of yeast strain BY4741 expressing either EYFP-Cub or Nyc-Cub showing that EYFP-Cub is in the cytoplasm and Nyc-Cub is localized to the plasma membrane. B. (Right) Western blot of membrane (M) and cytosolic (C) fractions from yeast strain NMY32 transfected with control plasmid (Alg5-Cub), untransfected cells or Nyc-Cub. These data indicate nyctalopin is membrane bound.

Sequence analyses of murine nyctalopin using seven different topology prediction programs (HMMTOP2.0, [25]; TMPred, [26]; TopPred2, [27]; SOSUI-Mp1, [28]; MemPype, [29]; TMHMM2.0, [30]; DAS, [31]) with the default parameters gave a variety of results (Table 1). It can be seen that there is no consensus with respect to the number and/or even the presence of transmembrane domains in murine nyctalopin. Five of the seven programs predicted a transmembrane domain at position 452–472 (TM1), three predicted a transmembrane domain at position 309–328 (TM2) and one predicted a single transmembrane domain at position 260–278 (TM3). Finally, DAS-TMfilter failed to identify any transmembrane domains. TMHMM2.0 has strong support for TM1 (probability of 0.9) and weaker support for TM2 probability (0.6). From these analyses, there may be as many as three transmembrane domains in murine nyctalopin (Figure S1). The most support is for TM1 (452–472), then TM2 (309–328), and there is a very weak support for TM3 (260–278) being a real transmembrane domain. TM3 also is located within the LRR domain, which is thought to be an interaction domain and therefore unlikely to contain a transmembrane region.

Given the computational and experimental (see below) support for the TM1 domain in murine nyctalopin, whereas human is GPI-anchored, we examined the predicted mode of anchoring for several other species. We used TMHH2.0 [30], big-PI [32] and MemPype [29] prediction programs in an attempt to identify the most likely location of transmembrane and GPI anchor sites, if present, in nyctalopin sequences from several species (Figure 1). These data showed that of the 9 mammals in the data set, the murine sequence has by far the most support for a transmembrane domain (TMHHM2.0, probability of ∼0.9) and rat had moderate (∼0.6) support for a transmembrane domain. The remaining species had very weak (<0.2) support for a transmembrane domain, although MemPype predicted a transmembrane domain in the rabbit sequence. Big-PI showed weak support for a GPI-anchor in all the sequences. MemPype predicted transmembrane domains in 3 species and GPI anchors in 8 species (Figure 1). Given human nyctalopin has been shown to be GPI anchored [7], it is clear that experiential data is required to confirm the methods by which nyctalopin is anchored in each species.

**Figure 3:**
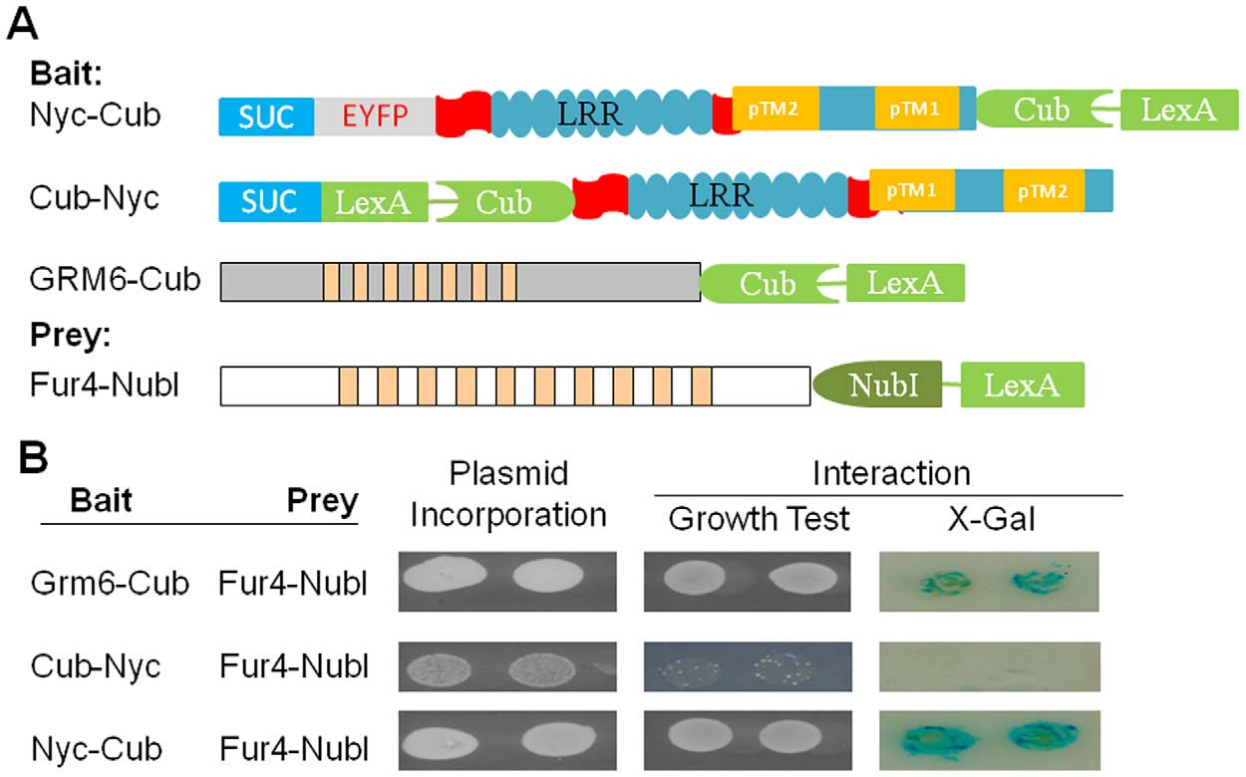
The N-terminus of nyctalopin is located in the extracellular space. A. Schematic diagrams of constructs used to determine the orientation of nyctalopin in the yeast membrane. Bait constructs use the yeast invertase (SUC) signal sequence. In the schematic, the blue rectangle represents the invertase signal sequence (SUC), the grey rectangle EYFP, the aqua ovals each LRR domain, the aqua rectangle with predicted transmembrane (TM) domain (orange rectangle) , the chevron represents the C-terminus of ubiquitin (Cub) and the green rectangle represents the artificial transcription factor (VP16LexA). Cub-Nyc has CubLexAVP16 inserted between the SUC signal sequence and nyctalopin. B. Membrane yeast two hybrid analysis of nyctalopin orientation in the membrane. Plasmid incorporation (column 1) is confirmed by growth on SD/-LW plates. When NubI and CubLexAVP16 are both localized to the cytoplasm, interaction occurs and supports growth on SD/-LWHA media and expression of β-galactosidase. Grm6-Cub is used as a positive control. The lack of growth when Cub-Nyc and Fur4-NubI are co-expressed indicates that the N-terminus of nyctalopin is not in the cytoplasm. Growth and expression of β-galactosidase when Nyc-Cub and Fur4-NubI are co-transformed indicate the C-terminus of nyctalopin is localized in the cytoplasm.

### Nyctalopin’s Leucine Rich Repeat (LRR) Domain is Oriented into the Extracellular Matrix

To examine the membrane topology of murine nyctalopin, we used a membrane split ubiquitin yeast two hybrid system (MYTH). The MYTH system is based on the ability of the N- and the C-terminal domains of ubiquitin, referred to as NubI and Cub, respectively, to interact even when they are two separate peptides [33], [34], [35]. Bait proteins are fused to Cub, which also is fused to the LexAVP16 transcription factor. Prey proteins are fused to either the NubI or its mutated form; NubG; which has an I13G mutation. This substitution makes the reconstitution of functional ubiquitin dependent on the interaction of the bait and prey fusion proteins.When Cub-LexA-VP16 and NubI are present in the cytoplasm, the two ubiqutin domains interact, reconstituting ubiquitin. The reconstituted ubiquitin is recognized by ubiquitin proteases (only present in the cytoplasm), which releases LexAVP16 that translocates to the nucleus and activates transcription of target selectable and/or marker genes.

To determine if nyctalopin was inserted into the membrane in yeast we transformed yeast with EYFP-Cub, Nyc-Cub or Alg5-Cub (a known membrane bound protein, [36]) (Figure 2A) and determined if they were membrane bound. Immunohistochemical analysis of the expression pattern showed that EYFP-Cub is cytoplasmic and Nyc-Cub is localized to the cell surface (Figure 2B). To confirm this biochemically, we isolated membrane and cytoplasmic fractions of yeast transfected with Alg5-Cub (membrane bound positive control) and Nyc-Cub. As with the Alg5 control, nyctalopin is also localized to the membrane fraction (Figure 2B).

**Figure 4:**
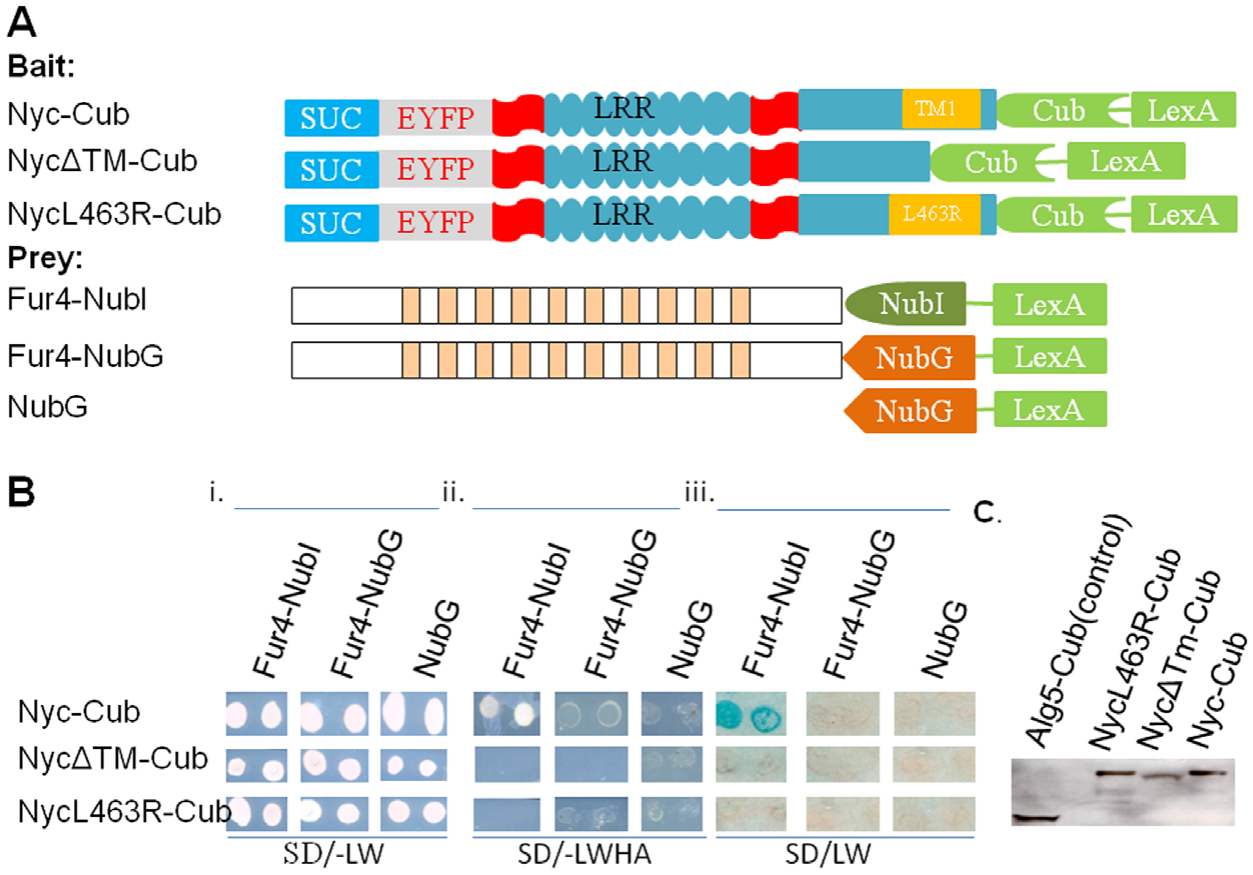
Genetic analysis shows murine nyctalopin has a single transmembrane domain. A. Schematic diagram of bait and prey constructs used to dissect the topology of nyctalopin. In the schematic, the blue rectangle represents the invertase signal sequence (SUC), the grey rectangle EYFP, the aqua ovals each LRR domain, the aqua rectangle with predicted transmembrane (TM) domain (orange rectangle) the C-terminus of nyctalopin, the chevron the C-terminus of ubiquitin (Cub) and the green rectangle the artificial transcription factor VP16LexA. NycΔTM-Cub has amino acids 455–476 (containing TM1) deleted from nyctalopin. NycL463R-Cub has a base substitution that creates a leucine to arginine substitution at position 463 of nyctalopin. Bi. Shows that all bait and prey plasmids were present in the NMY32 yeast strain and supported growth on the selective SD/-LW plates. Bii. The growth tests for interaction of bait and prey fusion proteins when the transformants are grown on SD/-LWHA plates. Biii. An X-gal assay for expression of β-galactosidase confirming the interaction in Bii. These results support the conclusion that only the Nyc-Cub/Fur4-NubI combination interact, indicating that there is a transmembrane domain in nyctalopin and that the C-terminus of nyctalopin is cytoplasmic. C. Western blot showing truncated transmembrane and L463R mutants are expressed in NMY32 yeast strain.

After confirming that nyctalopin was properly targeted to the membrane in yeast cells, we explored its orientation using the MYTH system. As a positive control, we attached Cub-VP16LexA to the C-terminus of the G-protein coupled receptor, GRM6, the C-terminus of which is localized to the cytoplasm (Figure 3A). To determine the orientation of nyctalopin, we attached the Cub-VP16LexA-domain to either the C-terminus (Nyc-Cub) or the N-terminus (Cub-Nyc) of nyctalopin (Figure 3A). To generate a prey protein with known sub-cellular location, we attached NubI to the C-terminus of the yeast plasma membrane protein; uracil permease (Fur4) (Figure 3A). Fur4 is known to orient in the plasma membrane with both C- and N-termini in the cytoplasm [37]. Co-transformation of Grm6-Cub and Fur4-NubI shows that Cub and NubI interacts, as indicated by growth on SD-LWHA selective plates as well as giving a positive X-gal assay (Figure 3B, row 1). In contrast, only when Cub-VP16LexA was attached to the C-terminus of nyctalopin was interaction detected (Figure 3B, row 2 compared to row 3). These data show that nyctalopin is oriented such that the C-terminus is intracellular. Nyctalopin also does not have two transmembrane domains, because if this was the case both C-and N-termini would be either intra- or extra-cellular. If both termini were intracellular, Nyc-Cub and Cub-Nyc would both interact with NubI. Similarly if both were extracellular neither would interact. The results (Figure 3B) do not support either conclusion, indicating nyctalopin does not contain two transmembrane domains.

Computer predictions indicated the most likely location of the transmembrane domain was from amino acid 452–472. To examine this prediction, we made a deletion mutant (NycΔTM1)-Cub and also substituted a hydrophilic arginine for leucine at position 463 within the predicted TM1 (NycL463R-Cub) (Figure 4A). Using the membrane prediction program TMHMM this L463R mutation in nyctalopin reduces the probability of a membrane domain being present from ∼0.9 to 0.1. If TM1 is the only transmembrane domain, then both mutant proteins should be secreted into the lumen of the ER. This will prevent interaction of the attached Cub with Fur4-NubI, which is localized to the plasma membrane with both termini in the cytoplasm. The bait plasmids, Nyc-Cub and the two mutant constructs, were each co-transfected with three different prey vectors (Figure 4B). Fur4-NubI was used to test for membrane insertion and orientation. Fur4-NubG was used to test for specificity of interaction between Cub and NubI, and a control vector expressing only NubG was used to test for self-activation (Figure 4A, Prey). Figure 4B shows that all plasmids are incorporated into the yeast and express the selectable markers. Only the parental Nyc-Cub/Fur4-NubI combination show any growth or β-galactosidase expression on the quadruple drop out plates (Figure 2B). These data indicate that the mutant Nyc-Cub constructs are not inserted into either the ER or plasma membrane, or released into the cytoplasm. This conclusion assumes that the deletion and mutation constructs were expressed. This was confirmed by analyzing yeast extracts by western blot, using antibodies to LexA. In all cases the bait protein was present at the expected size and at quantitatively similar expression levels (Figure 4C).

The failure of the combination of Nyc-Cub/NubG to grow or express β-galactosidase suggests that murine nyctalopin is not GPI anchored. If it was, the carboxy Cub-VP16LexA would have been cleaved and this alone would have supported growth and β-galactosidase expression.

### Murine Nyctalopin does not Homodimerize in Yeast

**Figure 5:**
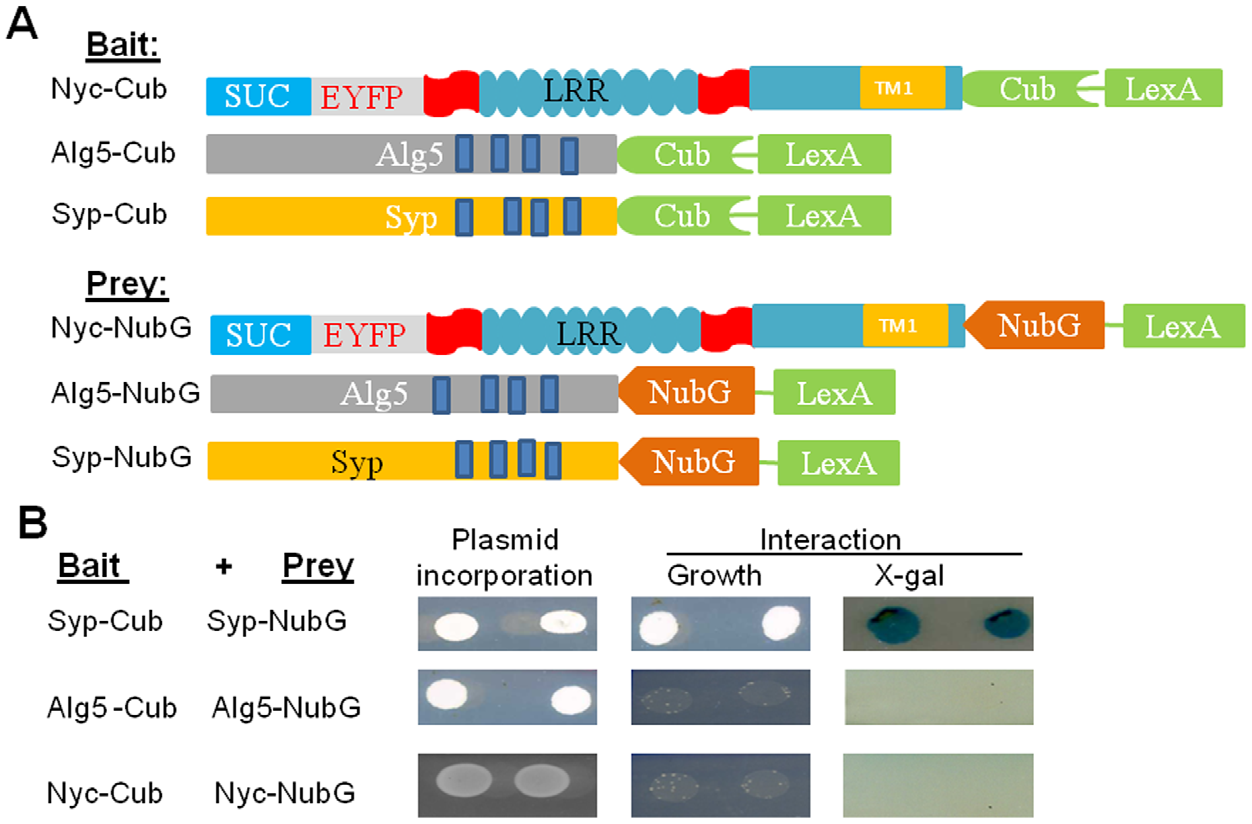
Nyctalopin does not form homo-dimers in yeast. A. Schematic of bait and prey constructs used. The components of nyctalopin are as described in Fig. 1 and 2. Alg5, Asparagine-linked glycosylation 5, is a yeast ER membrane bound protein with both the C-terminus and N-terminus in the cytoplasm. Syp-Cub and Syp-NubG are bait and prey constructs with synaptophysin, which is known to dimerize, and is used as positive control. B. Growth indicating incorporation of both bait and prey plasmids into yeast. Interaction was determined by growth on SD/-LWHA media and expression of β-galactosidase. These data show that nyctalopin does not form dimers in this system.

Several members of the SLRP family including decorin, opticin, and biglycan have been shown to dimerize through their LRR domains [22], [24], [38]. To determine if nyctalopin could dimerize in yeast, we cloned full length nyctalopin with its signal sequence replaced by the *S. cerevisiae* invertase signal sequence (SUC) into the prey vector pDL2N-SUC. This fuses NubG to the C-terminus of nyctalopin (Nyc-NubG) (Figure 5A). We used synaptophysin-Cub (Syp-Cub) and synaptophysin-NubG (Syp-NubG), which have been shown to form dimers using the MYTH system [39] as a positive control. The Alg5-Cub and Alg5-NubG combination was used as a negative control. Interactions between bait and prey vectors were tested by growth on SD-LWHA plates as well as the X-gal assay. The synaptopysin bait/prey combination showed both growth and expression of β-galactosidase as expected. However, neither the Alg5 nor the nyctalopin bait and prey combinations showed either growth or expression of β-galactosidase (Figure 5B). These data indicate that nyctalopin does not form dimers in yeast.

**Figure 6:**
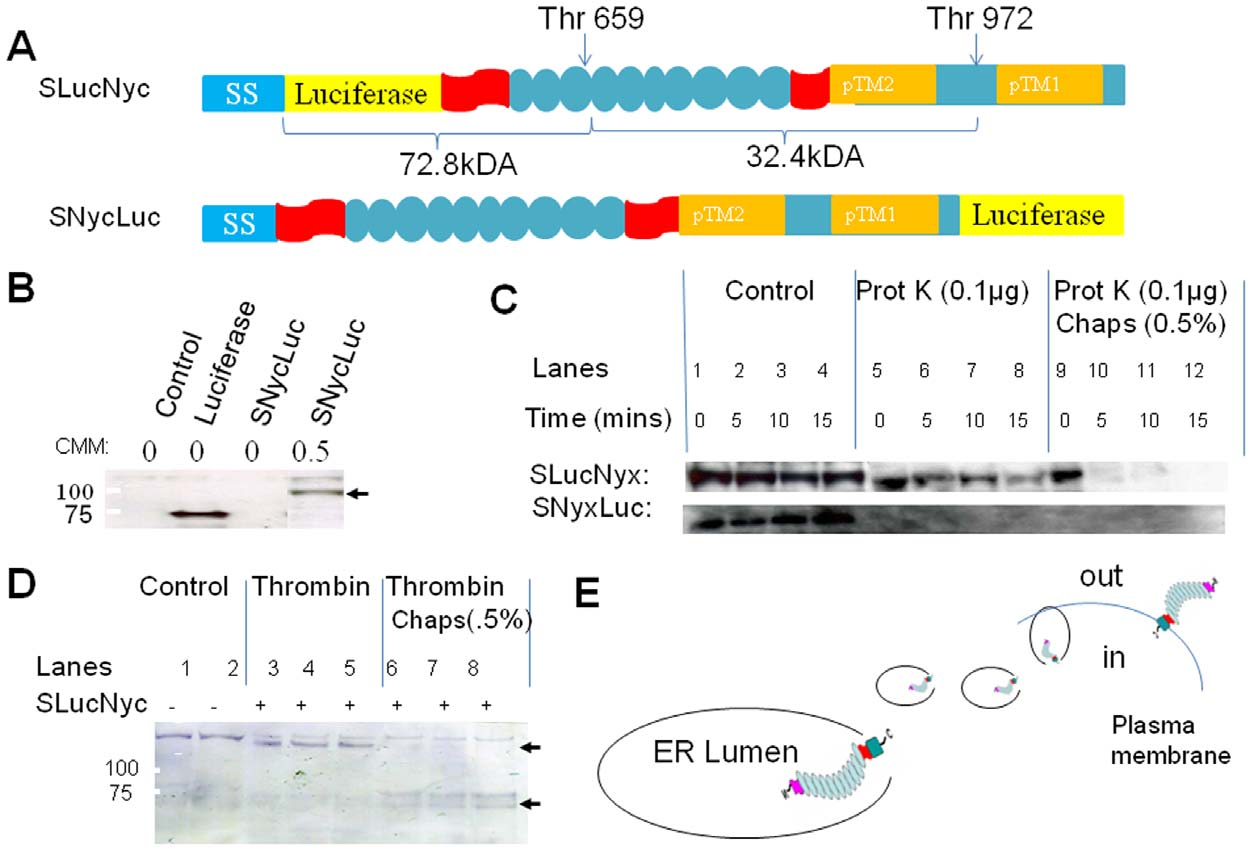
The N-terminus of nyctalopin is in the lumen of the ER. A. Schematic of constructs used to determine the orientation of nyctalopin in the membrane of the endoplasmic reticulum. SLucNyc (113 kDa) has luciferase (yellow rectangle) inserted after the murine nyctalopin signal sequence (SS). SNycLuc has luciferase attached to the C-terminus of full length nyctalopin. The arrows indicate the two thrombin (Thr) cleavage sites. Thrombin cleavage will generate 72.8 kDa and 34.4 kDa peptides, however, only the 72.8 kDa peptide will be labeled with biotinylated lysine and detected on a western blot. B. Western blot of *in vitro* transcription/translation reaction showing that without canine microsomal membranes (CMM), nyctalopin is not expressed. These data indicate co-translational processing and membrane insertion of nyctalopin in the ER. C. Expression of either SLucNyx or SNycLuc and treatment with proteinase K in the presence or absence of CHAPS. Lanes 1–4 indicate robust expression of full length nyctalopin. Lanes 5–8 show that SNycLuc but not SLucNyc is degraded by proteinase K, indicating the N-terminus of nyctalopin is in the ER lumen and therefore protected from degradation. Addition of CHAPS (0.5%) (lanes 9–12) disrupts the membranes and results in proteinase K digestion of both nyctalopin fusion proteins. D. Western blot of lysates from *in vitro* translation reactions, control (lanes 1–2), or when SLucNyc was included and after thrombin digestion alone (lanes 3–5) or thrombin digestion in the presence of 0.5% CHAPS (Lanes 6–8). Disruption of microsomal membranes with CHAPS allows cleavage of the fusion protein demonstrating that the N-terminus of nyctalopin is protected, and is therefore located in the lumen of the ER. Note the disappearance of the 113 kDa bands and the appearance of the 72 kDa band. E. Schematic showing a model of the orientation of nyctalopin in the ER and it’s subsequent disposition on the plasma membrane.

### The LRR Domain of Nyctalopin is Extracellular

One of the limitations of the topology experiments in yeast is the fact that to obtain optimal expression of murine proteins, we had to replace the nyctalopin signal sequence with the *S. cerevisiae* invertase signal (SUC) sequence. This could potentially alter the topology of nyctalopin. To provide additional support for the proposed topology we used a mammalian based *in vitro* transcription/translation system to evaluate post translational processing directly. Detection of the translated proteins in the system is based on incorporation of biotinylated lysine-tRNA (transcend tRNA), which is incorporated by the addition of precharged epsilon-labeled tRNA. This allows the use of streptavidin conjugated horseradish peroxidase (Strep-HRP) or streptavidin conjugated alkaline phosphatase (Strep-AP) for detection of newly synthesized protein on western blots. Nyctalopin only contains 2 lysines, therefore luciferase, which contains 40 lysines, was inserted after the nyctalopin signal sequence (SS) to increase detection sensitivity (SLucNyc) (Figure 6A). This should not disrupt function because insertion of EYFP at the same location generated a fully functional fusion protein [3]. A second vector with luciferase fused to the C-terminus of nyctalopin (SNycLuc), also was constructed (Figure 6A). A plasmid containing only luciferase was used as a positive control.

First, we determined if nyctalopin is co-translationally processed by translating nyctalopin in the presence or absence of canine microsomal membrane (CMM). Nyctalopin is predicted to contain a signal sequence and with the N-terminus located in the lumen of the ER. This conclusion was supported by the observation that to obtain robust translation, addition of CMM was required (Figure 6B, last lane). We next determined the orientation of nyctalopin using proteinase K digestion. If the N-terminus of nyctalopin is in the lumen of the microsomes as predicted, it should be protected from proteinase K digestion. Both SLucNyc and SNycLuc were tested in the coupled translation/transcription assay, with subsequent digestion by proteinase K. Figure 6C shows that there is no digestion of SLucNyc, whereas SNycLuc was digested by proteinase K. Protection for SLucNyc was lost when CHAPS was added. These results indicate that the N-terminus of nyctalopin is protected: and therefore is localized to the lumen of the microsomes and the C-terminus of nyctalopin is in the cytoplasm.

The experiment using proteinase K shows that the N-terminus is protected from digestion. However, after sufficient time proteinase K can digest all proteins even in the absence of CHAPS. To independently confirm that nyctalopin is oriented with the N-terminus in the lumen of the ER and the C-terminus in the cytoplasm, we used a second protease, thrombin, to examine membrane orientation of nyctalopin after *in vitro* translation. There are two thrombin cleavage sites in SLucNyc, one at position 659 and the other at position 972 (Figure 6A). The yeast two hybrid and proteinase K protection data above suggest that the N-terminus containing luciferase should be in the lumen of the microsomes and therefore protected from thrombin cleavage. Figure 6D shows that in the presence of intact microsomes, SLucNyc is protected from thrombin digestion. However, when the microsomes are disrupted by adding CHAPS, the protection is lost as can be seen by the generation of the 72.8 kDa cleavage product (Figure 6D, bottom arrow) and the disappearance of the 113 kDa SlucNyc band (Figure 6D, upper arrow). These data indicate that nyctalopin is oriented with the LRR domain in the lumen of the ER, which will result in this domain being present in the extracellular space once mature vesicles containing nyctalopin are fused with the plasma membrane (Figure 6E).

## Discussion

In the dark, photoreceptors release glutamate tonically into the synaptic cleft. The glutamate released binds to the metabotropic glutamate receptor (GRM6) on DBCs [40], [41] or the AMPA/kainate receptors on hyperpolarizing bipolar cells [42], [43], [44]. Glutamate binding to the GRM6 receptor activates a G-protein signal transduction cascade that closes a non-selective cation channel on the depolarizing bipolar cells [45], [46], [47], recently identified as TRPM1 [48], [49], [50]. When there is an increase in light intensity, glutamate release from photoreceptors is decreased, which leads to reduced GRM6 receptor activity, inactivation of the G-protein cascade and opening of the TRPM1 channel, causing depolarization of the DBCs. The depolarization is seen in an electroretinogram as a positive going b-wave. Defects in this signaling cascade result in loss of the ERG b-wave, and a class of human diseases called complete congenital stationary night blindness (CSNB) or CSNB1. Previous data showed that mutations in nyctalopin predicted to cause a loss of nyctalopin in humans and mouse, result in the absence of b-wave in the ERG [1], [2], [6], indicating signaling between the GRM6 receptor and TRPM1 is defective. Our topological analyses of nyctalopin show that the entire LRR domain is in the extracellular space, suggesting that this domain cannot be directly involved in the intracellular trimeric G-protein signaling cascade. Murine nyctalopin is predicted to have as many as 5 amino acids within the cytoplasm; so in theory these could interact with other components of the cascade. However, given human nyctalopin is thought to be GPI anchored, and therefore lacking this region, we suggest it is unlikely that the intracellular amino acids in murine nyctalopin have any function. Experiments such as truncation of these amino acids and subsequent functional analyses would, however, be needed to confirm this point. Further, our recent data and that of others indicate that nyctalopin interacts directly with TRPM1 [51], [52] and is required to localize TRPM1 to the tips of DBCs.

LRR domains have been shown to be involved in protein-protein interactions in several SLRP family members [4], [53], [54], [55] and other proteins (for review see ref [56]). That this domain is critical to function in nyctalopin is highlighted by the fact that mutations in the LRR domain of nyctalopin in humans cause CSNB1 [1], [2]. These data, combined with our observation that nyctalopin is required for the localization of TRPM1 to the dendritic tips of DBCs [52], suggest several possible mechanisms of action. Functional TRP channels are homo or hetero tetramers (for reviews see [57], [58]). Therefore, it is possible that nyctalopin is required for stabilization of the tetrameric structure in the membrane. In support of this, decorin, which is a close relative of nyctalopin, has been shown to interact with epidermal growth factor (EGF) receptor, causing dimerization and subsequent internalization of the EGF [59], [60]. The detailed mechanism of action of nyctalopin will require detailed dissection of the protein protein interaction domains as well as the predicted glycosylation of nyctalopin, although its functional form does not appear to be a dimer, at least in yeast.

In this report we have shown that murine nyctalopin is a transmembrane protein. In contrast human nyctalopin is anchored to the membrane by a GPI moiety [7]. Further experimental analyses will be required to determine which combinations of amino acid substitutions in murine nyctalopin compared to human nyctalopin result in a transmembrane anchor. These findings do suggest that how nyctalopin is anchored to the membrane is not critical to its function; rather it may be the structure of the extracellular domain. Future studies to elucidate the proteins with which nyctalopin interacts, and the critical regions of nyctalopin involved in these interactions will likely shed light not only on nyctalopin , but also the role of LRR domains in many other proteins.

## Methods

### Yeast Strains and Growth Media

Yeast strains used in this study are NYM32 (MATa his3 Δ 200 trp1–901 leu2–3,112 ade2 LYS2::(lexAop)_4_-HIS3 URA3::(lexAop)_8_-lacZ (lexAop)_8_-ADE2 GAL4)) and BY4741 (MATa; his3Δ1; leu2Δ0; met15Δ0; ura3Δ0) strains. The growth media used is synthetic media lacking leucine, tryptophan (SD/-LW), and leucine, tryptophan, histidine and adenine (SD/-LWHA) (Clontech, Moutain View, CA). All transformations were done using the lithium chloride method (Clontech, Moutain View, CA).

### Ethical Statement

This study was carried out in strict accordance with the recommendations in the Guide for the Care and Use of Laboratory Animals of the National Institutes of Health. The protocol was approved by the Institutional Animal Care and Use Committee of the University of Louisville (Protocol Number 10065). Retinas were removed for RNA isolation after euthanasia of adult C57BL/6J mice by carbon dioxide inhalation followed by cervical dislocation, as recommended by the American Veterinary Medical Association.

### Plasmid Constructs for Topology Experiments

Full length cDNA representing nyctalopin was obtained by PCR using cDNA synthesized from mRNA isolated from retinas of adult C57BL/6J mice. Vectors were constructed using infusion cloning techniques (Clontech, Moutain View, CA). For membrane topology experiments, the yeast membrane two hybrid (MYTH) system (Dualsystems, Grabenstrasse, Switzerland) bait vectors, pCCW-SUC, pNCW, pAI-Alg5, and prey vector, pDL2Nx, were used. Enhanced yellow fluorescent protein (EYFP) cDNA was cloned into the *Pst*I site of pCCW-SUC yielding EYFP-Cub. Nyc-Cub was made by inserting the nyctalopin cDNA encoding amino acids 23–476 of nyctalopin into the *Sfi*I site of Eyfp-Cub, which fused the C-terminus of nyctalopin to the C-terminal domain of ubiquitin (Cub) and the synthetic transcription factor (LexAVP16). To generate Cub-Nyc, the signal sequence of the yeast invertase gene was cloned into the *Xba*I site of pNCW. Nyctalopin cDNA encoding amino acids 23–476 was cloned into the *Pst*I site of pNCW. Grm6-Cub was made by cloning a full length Grm6 cDNA without its signal sequence into the *Sfi*I site of pCCW-SUC. Fur4-NubI was made by digesting pAI-Alg5 with *Spe*I and *Cla*I restriction enzymes to remove the *Alg5* gene and inserting the *Fur4* gene. Alg5-NubG was made by digesting pAI-Alg5 with *Age*I and *Xho*I, which removes the N-terminus of ubiquitin (NubI) from the pAI-Alg5 vector. NubI was replaced by a PCR product in which the substitution, I13G was introduced to produce Alg5-NubG. Fur4-NubG was made by cloning the *Fur4* gene into the pDL2Nx prey vector.

Nyctalopin dimerization was assayed by cloning full length nyctalopin cDNA into the *SfiI* site of pDL2Nx, creating Nyc-NubG. As a positive control, cDNA encoding synaptopysin (Syp) was cloned into the *Sfi*I site of the pCCW-Ste and pDL2N-Ste, creating Syp-Cub and Syp-NubG, respectively.

For mutagenesis experiments, NycΔTM3-Cub was created as a deletion variant of Nyc-Cub. This construct had nyctalopin amino acids 455–476 deleted. NycL463R-Cub had a single amino acid substitution in predicted transmembrane domain 1 (TM1).

### Constructs used in the *in vitro* Transcription/Translation Experiments

SNycLuc was made by inserting a full length nyctalopin cDNA into the *BamH*I site of the T7 Luciferase Control vector (Promega, Madison, WI). This arrangement fused luciferase to the C-terminus of nyctalopin. For the SLucNyc, the T7 luciferase vector was re-engineered so that the nyctalopin signal sequence was fused to luciferase, which was fused to amino acids 23–476 of nyctalopin. This arrangement put luciferase at the N-terminus of nyctalopin between the nyctalopin signal sequence (amino acids 1–22) and the rest of nyctalopin (amino acids 23–476).

### Colony Lift Assay

To determine if β-galactosidase was expressed, a single yeast colony was picked from a selection plate, streaked onto a new synthetic dropout (SD) plate (SD/-LW) and incubated for 2 to 3 days at 30°C. A Whatman 3 MM filter paper was placed directly onto the agar plate in contact with the yeast colonies for 10 min, carefully removed and transferred into liquid nitrogen for 2 min to lyse the cells. The filter paper was thawed for 5 min at room temperature, then incubated in freshly prepared agarose X-gal mix (0.5% agarose in 1xPBS, containing 0.1 mg/ml X-gal). Incubation at room temperature was for either 8 hours or until a blue color developed.

### Immunohistochemistry

Yeast were grown to mid-log (OD_600_ = 0.5–0.75) and fixed in PBS containing 2% glucose, 4 mM EGTA, and 7.4% formaldehyde, for 1 hour. The cells were recovered by centrifuging at 3000×g for 5 min and washed twice in PS-Buffer (0.1 M NaPO_3_, pH 6.6, 1.2 M sorbitol). To make spheroplasts, cells were incubated for 15 min in PS-buffer containing 7.1 µM β-mercaptoethanol and zymolyase (50 U/ml) at 37°C. After two washes in PS-buffer, cells were added to polylysine coated glass microscope slides, incubated at room temperature for 15 min and washed in PS-buffer three times. The cells were permeabilized in P-buffer (10 mg/ml of BSA, 0.5% SDS in PBS) for 15 min, washed ten times in PBS/BSA Buffer (10 mg/ml BSA in PBS) and then blocked for 1 hour in this same buffer. At the end of blocking, cells were washed five times in PBS/BSA buffer by applying the buffer and aspirating. To stain cells, Alexa 488 conjugated anti-GFP antibody (Cat #: A2131, Invitrogen, Carlsbad, CA) was diluted 1∶1000 in PBS/BSA and incubated overnight in a moist chamber. Slides were washed ten times with PBS/BSA-Buffer followed by three times in PBS, then allowed to air dry at room temperature before cover slipping. Images were taken on an Olympus FV300 Confocal Microscope using a 60x objective and images were processed using Flowview software.

### *In vitro* Translation and the Protection Assays

*In vitro* transcription and translation were done according to the manufacturer’s protocol (Promega, Madison, WI, Cat # TM035). Plasmid DNA (2 μg) was added to each translation reaction. To maximize the translation of membrane proteins, canine microsomal membranes (Cat # Y4041 Promega, Madison, WI) were added to some samples. After 90 min incubation at 30°C, 2 µl of the translation mix was added to 13 µl of SDS sample buffer and heated at 70°C for 10 min. Samples were resolved on 4–12% NOVEX gradient PAGE gels (Invitrogen, Calsbad, CA).

To determine the orientation of proteins in the membrane, 10 µl of translation product was incubated with or without 0.1 µg of proteinase K (20 mg/ml). Samples were incubated on ice for 5, 10, 15, 20 min with or without the addition of Chaps (0.5%).

The thrombin cleavage mix contained; 1×thrombin cleavage buffer, 10 µg of total protein lysate, and thrombin (50 U) in a final volume of 50 µl. Samples were incubated at 20°C for 16 hours. 20 µl of the digested samples were added to 20 µl of SDS buffer, heated at 70°C and resolved by PAGE on 4–12% NOVEX gradient gels.

## Supporting Information

Figure S1. **Tertiary structure of murine nyctalopin and theoretical orientation.** A. The convex side of nyctalopin consists of parallel β-sheets and the concave side α-helices. The β-sheets and α-helices are folded to form 11 tandem leucine rich repeats, which are capped at the N- and C-termini by cysteine rich repeats. The N-terminus has a predicted signal sequence and the C-terminus has one or more predicted transmembrane domains. B. Possible orientations of nyctalopin dependent on whether there are three (I), two (II and III) one (IV) or no (V) transmembrane domains in nyctalopin.
(TIF)

## References

[1] Bech-Hansen NT, Naylor MJ, Maybaum TA, Sparkes RL, Koop B (2000). Mutations in NYX, encoding the leucine-rich proteoglycan
nyctalopin, cause X-linked complete congenital stationary night
blindness.. Nat Genet.

[2] Pusch CM, Zeitz C, Brandau O, Pesch K, Achatz H (2000). The complete form of X-linked congenital stationary night
blindness is caused by mutations in a gene encoding a leucine-rich repeat
protein.. Nat Genet.

[3] Gregg RG, Kamermans M, Klooster J, Lukasiewicz PD, Peachey NS (2007). Nyctalopin expression in retinal bipolar cells restores visual
function in a mouse model of complete X-linked congenital stationary night
blindness.. J Neurophysiol.

[4] Schaefer L, Iozzo RV (2008). Biological functions of the small leucine-rich proteoglycans:
from genetics to signal transduction.. J Biol Chem.

[5] Takahashi N, Takahashi Y, Putnam FW (1985). Periodicity of leucine and tandem repetition of a 24-amino acid
segment in the primary structure of leucine-rich alpha 2-glycoprotein of
human serum.. Proc Natl Acad Sci U S A.

[6] Gregg RG, Mukhopadhyay S, Candille SI, Ball SL, Pardue MT (2003). Identification of the gene and the mutation responsible for the
mouse nob phenotype.. Invest Ophthalmol Vis Sci.

[7] O’Connor E, Eisenhaber B, Dalley J, Wang T, Missen C (2005). Species specific membrane anchoring of nyctalopin, a small
leucine-rich repeat protein.. Hum Mol Genet.

[8] Zeitz C, Scherthan H, Freier S, Feil S, Suckow V (2003). NYX (nyctalopin on chromosome X), the gene mutated in congenital
stationary night blindness, encodes a cell surface protein.. Invest Ophthalmol Vis Sci.

[9] Saraogi I, Shan SO (2011). Molecular mechanism of co-translational protein targeting by the
signal recognition particle.. Traffic.

[10] Egea PF, Stroud RM, Walter P (2005). Targeting proteins to membranes: structure of the signal
recognition particle.. Current Opinion in Structural Biology.

[11] Denzer AJ, Nabholz CE, Spiess M (1995). Transmembrane orientation of signal-anchor proteins is affected
by the folding state but not the size of the N-terminal
domain.. EMBO J.

[12] Dalbey RE, Wang P, Kuhn A (2011). Assembly of bacterial inner membrane proteins.. Annu Rev Biochem.

[13] Sakaguchi M, Tomiyoshi R, Kuroiwa T, Mihara K, Omura T (1992). Functions of signal and signal-anchor sequences are determined by
the balance between the hydrophobic segment and the N-terminal
charge.. Proc Natl Acad Sci U S A.

[14] Wahlberg JM, Spiess M (1997). Multiple determinants direct the orientation of signal-anchor
proteins: the topogenic role of the hydrophobic signal
domain.. J Cell Biol.

[15] Dancourt J, Barlowe C (2010). Protein sorting receptors in the early secretory
pathway.. Annu Rev Biochem.

[16] Appenzeller-Herzog C, Hauri HP (2006). The ER-Golgi intermediate compartment (ERGIC): in search of its
identity and function.. J Cell Sci.

[17] De Matteis MA, Luini A (2008). Exiting the Golgi complex.. Nat Rev Mol Cell Biol.

[18] Windisch JM, Marksteiner R, Schneider R (1995). Nerve growth factor binding site on TrkA mapped to a single
24-amino acid leucine-rich motif.. J Biol Chem.

[19] Meadows LA, Gell D, Broadie K, Gould AP, White RA (1994). The cell adhesion molecule, connectin, and the development of the
Drosophila neuromuscular system.. J Cell Sci 107 ( Pt.

[20] Kobe B, Deisenhofer J (1996). Mechanism of ribonuclease inhibition by ribonuclease inhibitor
protein based on the crystal structure of its complex with ribonuclease A. J
Mol Biol.

[21] Liu J, Laue TM, Choi HU, Tang LH, Rosenberg L (1994). The self-association of biglycan from bovine articular
cartilage.. J Biol Chem.

[22] Scott PG, Grossmann JG, Dodd CM, Sheehan JK, Bishop PN (2003). Light and X-ray scattering show decorin to be a dimer in
solution.. J Biol Chem.

[23] Scott PG, McEwan PA, Dodd CM, Bergmann EM, Bishop PN (2004). Crystal structure of the dimeric protein core of decorin, the
archetypal small leucine-rich repeat proteoglycan.. Proc Natl Acad Sci U S A.

[24] Le Goff MM, Hindson VJ, Jowitt TA, Scott PG, Bishop PN (2003). Characterization of opticin and evidence of stable dimerization
in solution.. J Biol Chem.

[25] Tusnady GE, Simon I (2001). The HMMTOP transmembrane topology prediction
server.. Bioinformatics.

[26] Hofmann K, Stoffel W (1993). TMBASE - A database of membrane spanning protein
segments.. Biol Chem Hoppe-Seyler.

[27] Claros MG, von Heijne G (1994). TopPred II: an improved software for membrane protein structure
predictions.. Comput Appl Biosci.

[28] Hirokawa T, Boon-Chieng S, Mitaku S (1998). SOSUI: classification and secondary structure prediction system
for membrane proteins.. Bioinformatics.

[29] Pierleoni A, Indio V, Savojardo C, Fariselli P, Martelli PL (2011). MemPype: a pipeline for the annotation of eukaryotic membrane
proteins.. Nucleic Acids Res.

[30] Krogh A, Larsson B, von Heijne G, Sonnhammer EL (2001). Predicting transmembrane protein topology with a hidden Markov
model: application to complete genomes.. J Mol Biol.

[31] Cserzo M, Wallin E, Simon I, von Heijne G, Elofsson A (1997). Prediction of transmembrane alpha-helices in prokaryotic membrane
proteins: the dense alignment surface method.. Protein Eng.

[32] Eisenhaber B, Bork P, Eisenhaber F (1999). Prediction of potential GPI-modification sites in proprotein
sequences.. J Mol Biol.

[33] Johnsson N, Varshavsky A (1994). Split ubiquitin as a sensor of protein interactions in
vivo.. Proc Natl Acad Sci U S A.

[34] Stagljar I, Korostensky C, Johnsson N, te Heesen S (1998). A genetic system based on split-ubiquitin for the analysis of
interactions between membrane proteins in vivo.. Proc Natl Acad Sci U S A.

[35] Stagljar I (2003). Finding partners: emerging protein interaction technologies
applied to signaling networks..

[36] Heesen S, Lehle L, Weissmann A, Aebi M (1994). Isolation of the ALG5 locus encoding the
UDP-glucose:dolichyl-phosphate glucosyltransferase from Saccharomyces
cerevisiae.. Eur J Biochem.

[37] Garnier C, Blondel MO, Haguenauer-Tsapis R (1996). Membrane topology of the yeast uracil permease.. Mol Microbiol.

[38] Scott PG, Dodd CM, Bergmann EM, Sheehan JK, Bishop PN (2006). Crystal structure of the biglycan dimer and evidence that
dimerization is essential for folding and stability of class I small
leucine-rich repeat proteoglycans.. J Biol Chem.

[39] Felkl M, Leube RE (2008). Interaction assays in yeast and cultured cells confirm known and
identify novel partners of the synaptic vesicle protein
synaptophysin.. Neuroscience.

[40] Nomura A, Shigemoto R, Nakamura Y, Okamoto N, Mizuno N (1994). Developmentally regulated postsynaptic localization of a
metabotropic glutamate receptor in rat rod bipolar cells.. Cell.

[41] Vardi N, Morigiwa K (1997). ON cone bipolar cells in rat express the metabotropic receptor
mGluR6.. Vis Neurosci.

[42] Brandstatter JH, Koulen P, Wassle H (1997). Selective synaptic distribution of kainate receptor subunits in
the two plexiform layers of the rat retina.. J Neurosci.

[43] Hack I, Peichl L, Brandstatter JH (1999). An alternative pathway for rod signals in the rodent retina: rod
photoreceptors, cone bipolar cells, and the localization of glutamate
receptors.. Proc Natl Acad Sci U S A.

[44] DeVries SH, Schwartz EA (1999). Kainate receptors mediate synaptic transmission between cones and
‘Off’ bipolar cells in a mammalian retina.. Nature.

[45] Nawy S, Jahr CE (1991). cGMP-gated conductance in retinal bipolar cells is suppressed by
the photoreceptor transmitter.. Neuron.

[46] Yamashita M, Wassle H (1991). Responses of rod bipolar cells isolated from the rat retina to
the glutamate agonist 2-amino-4-phosphonobutyric acid (APB).. J Neurosci.

[47] Shiells RA, Falk G (1990). Glutamate receptors of rod bipolar cells are linked to a cyclic
GMP cascade via a G-protein.. Proc Biol Sci.

[48] Shen Y, Heimel JA, Kamermans M, Peachey NS, Gregg RG (2009). A transient receptor potential-like channel mediates synaptic
transmission in rod bipolar cells.. J Neurosci.

[49] Morgans CW, Zhang J, Jeffrey BG, Nelson SM, Burke NS (2009). TRPM1 is required for the depolarizing light response in retinal
ON-bipolar cells.. Proc Natl Acad Sci U S A.

[50] Koike C, Obara T, Uriu Y, Numata T, Sanuki R (2010). TRPM1 is a component of the retinal ON bipolar cell transduction
channel in the mGluR6 cascade.. Proc Natl Acad Sci U S A.

[51] Cao Y, Posokhova E, Martemyanov KA (2011). TRPM1 forms complexes with nyctalopin in vivo and accumulates in
postsynaptic compartment of ON-bipolar neurons in mGluR6-dependent
manner.. J Neurosci.

[52] Pearring JN, Bojang P, Shen Y, Koike C, Furukawa T (2011). A role for nyctalopin, a small leucine-rich repeat protein, in
localizing the TRP melastatin 1 channel to retinal depolarizing bipolar cell
dendrites.. J Neurosci.

[53] Brandan E, Cabello-Verrugio C, Vial C (2008). Novel regulatory mechanisms for the proteoglycans decorin and
biglycan during muscle formation and muscular dystrophy.. Matrix Biol.

[54] Perrimon N, Bernfield M (2001). Cellular functions of proteoglycans–an
overview.. Semin Cell Dev Biol.

[55] Iozzo RV, Moscatello DK, McQuillan DJ, Eichstetter I (1999). Decorin is a biological ligand for the epidermal growth factor
receptor.. J Biol Chem.

[56] Bella J, Hindle KL, McEwan PA, Lovell SC (2008). The leucine-rich repeat structure.. Cell Mol Life Sci.

[57] Jiang LH (2007). Subunit interaction in channel assembly and functional regulation
of transient receptor potential melastatin (TRPM) channels.. Biochem Soc Trans.

[58] Cheng W, Sun C, Zheng J (2010). Heteromerization of TRP channel subunits: extending functional
diversity.. Protein Cell.

[59] Zhu JX, Goldoni S, Bix G, Owens RT, McQuillan DJ (2005). Decorin evokes protracted internalization and degradation of the
epidermal growth factor receptor via caveolar endocytosis.. J Biol Chem.

[60] Lee JC, Gimm JA, Lo AJ, Koury MJ, Krauss SW (2004). Mechanism of protein sorting during erythroblast enucleation:
role of cytoskeletal connectivity.. Blood.

