# Supplementary figures and images for: Topological Analysis of Small Leucine-Rich Repeat Proteoglycan Nyctalopin
Source: PLoS One. 2012 Apr 2;7(4):e33137. doi: 10.1371/journal.pone.0033137 (PMC3317652; doi:10.1371/journal.pone.0033137)

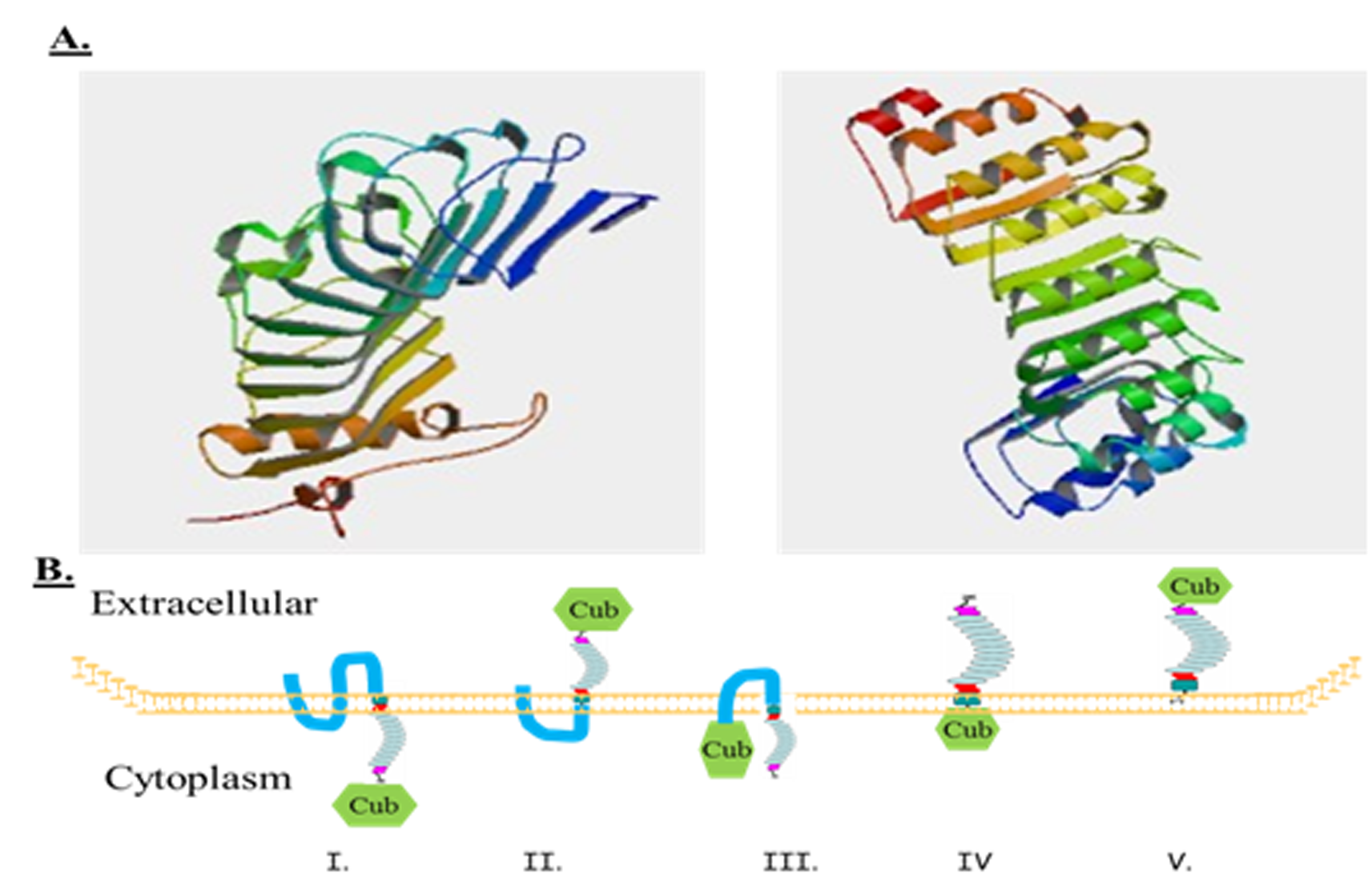

Supplement: Figure S1. — Tertiary structure of murine nyctalopin and theoretical orientation. A. The convex side of nyctalopin consists of parallel β-sheets and the concave side α-helices. The β-sheets and α-helices are folded to form 11 tandem leucine rich repeats, which are capped at the N- and C-termini by cysteine rich repeats. The N-terminus has a predicted signal sequence and the C-terminus has one or more predicted transmembrane domains. B. Possible orientations of nyctalopin dependent on whether there are three (I), two (II and III) one (IV) or no (V) transmembrane domains in nyctalopin. (TIF) [file pone.0033137.s001.tif]
